# Setting the Public Agenda for Online Health Search: A White Paper and Action Agenda

**DOI:** 10.2196/jmir.6.2.e18

**Published:** 2004-06-08

**Authors:** Liza Greenberg, Guy D'Andrea, Dan Lorence

**Affiliations:** ^1^Utilization Review Accreditation Commission Inc. (URAC)Washington DCUSA; ^2^Pennsylvania State UniversityUniversity Park PAUSA

**Keywords:** eHealth, Internet, information management, health services research, quality of health care, consumer participation, patient education

## Abstract

**Background:**

Searches for health information are among the most common reasons that consumers use the Internet. Both consumers and quality experts have raised concerns about the quality of information on the Web and the ability of consumers to find accurate information that meets their needs.

**Objective:**

To produce a national stakeholder-driven agenda for research, technical improvements, and education that will improve the results of consumer searches for health information on the Internet.

**Methods:**

URAC, a national accreditation organization, and Consumer WebWatch (CWW), a project of Consumers Union (a consumer advocacy organization), conducted a review of factors influencing the results of online health searches. The organizations convened two stakeholder groups of consumers, quality experts, search engine experts, researchers, health-care providers, informatics specialists, and others. Meeting participants reviewed existing information and developed recommendations for improving the results of online consumer searches for health information. Participants were not asked to vote on or endorse the recommendations. Our working definition of a quality Web site was one that contained accurate, reliable, and complete information.

**Results:**

The Internet has greatly improved access to health information for consumers. There is great variation in how consumers seek information via the Internet, and in how successful they are in searching for health information. Further, there is variation among Web sites, both in quality and accessibility. Many Web site features affect the capability of search engines to find and index them.

**Conclusions:**

Research is needed to define quality elements of Web sites that could be retrieved by search engines and understand how to meet the needs of different types of searchers. Technological research should seek to develop more sophisticated approaches for tagging information, and to develop searches that "learn" from consumer behavior. Finally, education initiatives are needed to help consumers search more effectively and to help them critically evaluate the information they find.

## Introduction

Searches for health information are among the most common reasons that consumers use the Internet. The Pew Internet & American Life Project (Pew) reported in 2003 that 80% of Americans with Internet access have used the Web to get health or medical information [1]. The Internet has transformed the ability of consumers to find health information and to connect with other individuals with similar interests. The Internet has been recognized as an important source of health information by the federal government, which established a series of goals relating to access and quality of information on the Internet in the Healthy People 2010 action plan [2].

Health information on the Internet can dramatically improve consumers' health-care and lifestyle choices. However, increased access to Web-based information has also raised concerns about the quality of information consumers are using, and the impact of this information [3]. Disparities in access to information have also become apparent. These factors suggest the need to better understand how consumers find health information on the Web, how to evaluate the quality of information retrieved, and how to help consumers to critically evaluate and manage information. These factors suggest the need to better understand how consumers find health information on the Web, how they evaluate the quality of information retrieved, and how to they could be helped to critically evaluate and manage information.

Research on health Web sites raised concerns about the quality of information on the Web [4]. A 2001 study by RAND for the California Healthcare Foundation showed that information on health Web sites is often incomplete or out of date [5]. This might be of little concern if consumers routinely consulted health-care professionals about the information. However, Pew found that 69% of consumers did not discuss the information they found with a doctor or nurse.

Many people use search engines to find the information they use to help make personal health decisions. Search engines and the Internet have vastly improved access to health information for many consumers. However, search processes and results vary considerably among search engines, and are not transparent to consumers. The criteria used to identify and rank health-related Web sites vary among search engines, and often is not apparent to consumers. Search results may be affected by the structure of content on health Web sites, consumer search terminology, and the use of paid placements by the search engine.

In short, research on health searches suggests that the process by which consumers locate health information on the Internet, and the evaluations they make regarding which Web sites to review are important variables in the quality of information they ultimately view and use. Improved understanding of factors influencing online searches will facilitate technical and educational approaches for maximizing quality and benefit of health searches.

## Methods

In 2003, URAC and Consumer WebWatch (CWW), a project of Consumers Union, carried out a project funded by the Robert Wood Johnson Foundation to examine factors influencing the results of online health searches and to develop an agenda for future research and development that would improve the results of health searches. We reviewed published literature and industry reports, and convened two stakeholder groups consisting of consumers, quality experts, search engine experts, researchers, health-care providers, informatics specialists and others.

### Literature Review Method

Our literature review was not exhaustive: its purpose was to provide a baseline understanding of consumer, Web site, quality measurement, and search engine factors that influence the results of searches for health information. We conducted a search of key terms in the Cumulative Index of Nursing and Allied Health Literature (CINAHL), Medline, PubMed, Expanded Academic ASAP, Lexis-Nexis, Proquest, Ingenta, and related databases in health care, information science, and computer science. The initial searches took place in early 2003, but citations were added as they were identified.

Where initial searches revealed poor topic coverage, associated reference lists, books and other media that were considered to inform the topic were included. The following search terms were included: Web-based, Web site, information quality, Web search, consumer health, eHealth, health information, search engine, information retrieval, information seeking. We also examined bibliographies of articles retrieved by electronic searches and solicited recommendations from members of the project advisory committee. We discontinued searching in specific topic areas when project staff believed we had adequately described current understanding of key issue areas.

### Methods for Convening Stakeholder Meetings

An open announcement about the project and recommendations from industry leaders helped identify interested stakeholders, and participants were selected by URAC and CWW with guidance from a project advisory committee. Not everyone invited was available to attend. We attempted to achieve a balance of different stakeholders at each meeting. Meetings were held in California and Washington, DC to facilitate participation.

The purpose of each stakeholder meeting was to review existing knowledge about results of consumer searches for health information, and to develop recommendations for additional research, technical improvements, and educational approaches needed to improve the results of online consumer searches for health information. Participants reviewed the summary recommendations presented in this article after the meeting and had the opportunity to comment, but were not asked to vote on or endorse the recommendations.

For the purposes of this project, we assumed that most searchers would prefer information that is accurate and reliable. These attributes are also components of effective health communication [2]. This was our working definition for quality Web sites. The perception of other elements that might be used to define quality, such as the site's reading level and comprehensiveness, will vary depending on the user and the user's needs at a given time.

## Results

### Results of Literature Review

#### How Consumers Use the Internet to Locate Health Information

An April 2003 report from the Pew Internet & American Life (Pew) report provided an overview of the US Internet consumer population [6]. The study found that Internet access has grown across-the-board, but that demographic gaps remain. A variety of factors continue to separate Internet users from non-users. Internet users tend to be younger and more affluent, and are more likely to be employed, white, well-educated, and to be suburban or urban residents.

Pew noted that consumers often overestimate their knowledge of the Internet and their ability to locate information. A 2002 analysis by Houston et al using Pew data noted a need to educate patients about searching for health information online and for tools to help them identify high quality information [7]. They also found that chronically ill Internet users were often relatively new to the Internet, but noted that they were more likely than those in good health to discuss findings with their physicians.

#### Consumer Search Strategies

A 2002 Pew Internet & American Life Project poll found that the typical health-information seeker usually starts searching for medical information at a general search site, not a medical site. Eighty-one percent of online health seekers start at a search engine or use the search function of a general portal such as the Yahoo home page, MSN, or AOL. Consumers visit two to five sites during an average visit and typically spend at least thirty minutes on a search [8].

Several studies have investigated behaviors consumers exhibit in retrieving and health information on the Internet and in assessing its quality. Eysenbach and Köhler, examining Web searchers in Germany, found that although search technique was often suboptimal, Internet users found the health information they were looking for relatively quickly [9]. A search optimization firm, iProspect, reports that users generally use the same search engine for all types of search requests. Users look at up to three pages of search results to determine relevance, and abandon a search if they do not find appropriate results in the first three pages. Users usually modify their query after abandoning an initial search, and may at that point change search engines [10]. These findings illustrate the importance of search engines to the process of retrieving health information. They imply a business rationale for search engines to ensure that health searchers locate what they want, since they may otherwise lose search traffic.

#### Comprehension, Literacy, and Access Issues

Searches are heavily influenced by the search terms used, even when the terms used are considered to be synonyms. Use of lay terminology for a health subject can result in unrelated or misleading information [11]. Berland et al concluded that accessing health information using search engines and simple search terms was not efficient because Web sites are inconsistent in their provision of key information, and because high reading levels are required to comprehend Web-based health information [4]. Also, the relevance of information located was often of limited value, which may have been due to terminology used in the original search phrase. Non-English speakers face challenges finding and reviewing information on the Internet. One Internet accessibility study for people with disabilities found that there are significant access barriers. Governmental and educational health-information Web sites were more accessible than other categories, such as Web portals and community sites [12].

#### Physician Responses to Internet Information

A study of physician views on online information found that physicians increasingly encounter patients who have conducted health searches. Use of the Internet by patients does appear to affect treatment processes: for example, many physicians reported having changed the treatment protocols they had initially planned as a result of consumer requests. Although most physicians believe the information their patients find is accurate, many believe that having to discuss this information with their patients decreases their efficiency and challenges their authority. Some are also concerned that the information may be inaccurate. The study concluded that quality of information on the Internet is critical, as it does influence both patient requests and physician treatment choices [13]. In an effort to steer patients to credible Web sites, some health-care organizations have begun to suggest ( "prescribe") credible Web sites to their patients in the course of their consultations [14].

#### Consumer Evaluation of Web Site Credibility

Experts and consumers use different criteria for evaluating the quality of Web sites. Eysenbach found that consumers assessing the credibility of a Web site primarily looked for the source, a professional design, a scientific or official touch, language, and ease of use. Study participants never checked any "about us" sections of Web sites, disclaimers, or disclosure statements. Very few participants noticed and remembered which Web sites visited [9]. A Consumer WebWatch (CWW) study of consumers reported findings similar to Eysenbach's: once people get to a site, they do not use rigorous criteria to assess the site's credibility. For example, they almost never referred to a site's privacy policy. The average consumer paid far more attention to the superficial aspects of a site, such as visual cues, than to its content. Nearly half of all consumers in the CWW study assessed the credibility of sites based in part on the appeal of the overall visual design, including layout, typography, font size, and color schemes. In comparison, a parallel group of health and finance experts were far less concerned about the surface aspects of these industry-specific types of sites and more concerned about the breadth, depth, and quality of a site's information [15].

### How Web Sites Influence Availability of Quality Health Information

#### Techniques for Conveying Information about Web Site Content

The structure of a Web site influences how information can be retrieved from the site by a search engine, as well as the usability of the site for consumers. Coding and structure of Web sites can facilitate retrieval by search engines or can pose a barrier to information retrieval. Coded information on a Web site is processed through the search engine algorithm, and determines whether and how the site is ranked in search returns. The same tags and codes that can be used to highlight information on a legitimate Web site may also be used by "spoofers" who try to lure traffic onto the site.

In general, Web sites can support retrieval of information on their pages by using metadata, metatags and keywords to guide search crawlers to important content. These codes provide a means for relaying information directly to the search engine. Keywords are recognized indicators of specific services or products that can be used to increase specificity of searches and help Web sites attract "qualified" traffic. One strategy for enhancing search rankings of quality Web sites is to code certain types of information for consistent retrieval by the search engine. Efforts are under way to implement metadata codes to support a "semantic Web." The semantic Web uses code to establish relationships between words to enable search engines to effectively understand intent, rather than simply identifying the presence of a search term [16].

#### Quality Indicators for Web Site Content

Eysenbach et al found wide-ranging differences in studies of the quality of health Web sites. There are significant variations in study methods and rigor, quality criteria, study population, and topic chosen. Operational definitions of quality are often inconsistent. As a result, the conclusions on quality of health-related Web sites vary widely. Eysenbach found that the most frequently used quality criteria include accuracy, completeness, readability, design, disclosures, and references provided [16].

Griffiths and Christensen evaluated the quality of Web-based information on treatment of depression to identify potential indicators of content quality, and to establish whether accountability criteria are indicators of quality [17]. They found that although the sites examined contained useful information, their overall quality was poor. Sites typically did not cite scientific evidence in support of their conclusions.

Researchers have also studied the correlation between popularity of Web sites and quality of content. Meric et al found that more popular breast cancer-related Web sites were more likely than less popular ones to contain information on ongoing clinical trials, results of trials, and opportunities for psychosocial adjustment. These characteristics were also associated with a higher number of links. More popular sites were more likely to provide updates on other breast cancer research, information on legislation and advocacy, and a message board service. Measures of quality such as display of authorship, attribution or references, currency of information, and disclosure did not differ between popular and less popular sites [18]. In similar findings, Kunst et al found that while there is a correlation between credibility features and accuracy of information, the association is relatively weak [19].

These findings suggest that additional research is needed to identify indicators of content quality, and to correlate consumer preferences to quality indicators. Sites that include content correlated with popularity may best meet the public's desire for health information. Current search algorithms may not be in agreement with quality clinical indicators and performance measures currently used throughout the health-care industry.

#### Codes of Conduct

A wide range of tools has been developed to assist site developers to produce good quality sites and for consumers to assess the quality of sites. Adherence to accepted codes could theoretically be used as a factor in searches. Ratings instruments include codes of conduct, quality labels, user guides, filters, and third party certification. The value of these tools is unclear: studies have demonstrated that consumers do not routinely seek out information on certifications or adherence to voluntary codes. However, it is assumed by many that such codes benefit consumers indirectly by influencing Web site behaviors and practices. For example, most standards require sites to implement privacy protections and disclosure of site information as consumer protections. No research has been done on the effect of compliance to a code of conduct on Web sites.

A number of organizations have developed quality criteria for health-related Web sites, some with verification and some completely voluntary. Voluntary, self-certifying standards have been developed by the eHealth Code of Ethics of the Internet Health Coalition [20], the American Medical Association [21] and the Health On the Net (HON) Foundation [22]. URAC has developed a health Web site accreditation program that involves independent verification of compliance with its standards. URAC accreditation includes review of the Web site by an external auditor [23].

#### Web Site Rating Instruments

Web site ratings could be potentially be used to inform searchers and search engines as well, if ratings could be clearly correlated to quality. Two common approaches to rating Web sites include expert ratings, and user (consumer) ratings. Gagliardi and Jadad conducted two evaluations of Web site rating tools, published in 1998 and 2002 respectively [24,25]. They concluded that ratings instruments tend to proliferate and disappear, and that few have been validated for direct correlation between standards and quality. Few provide details on how they were developed, or provide instructions for use, or information about the inter-observer reliability and construct validity of the measurements.

Kim et al reviewed published criteria for evaluating health-related information on the Web, and identified areas of criteria-based consensus [26]. They identified 29 published rating tools and journal articles that had explicit criteria for assessing health-related Web sites. The most frequently cited criteria were those dealing with content, design and aesthetics of site, disclosure of authors, sponsors or developers, currency of information (includes frequency of update, freshness, maintenance of site), authority of source, ease of use, and accessibility and availability.

A number of tools are available to guide users in evaluating information on the Web. Interactive user guidance systems can be used to assess characteristics of Web sites. Tools such as DISCERN and QUICK allow Web site users to assess Web site credibility by responding to a series of questions [27]. Other organizations such as the National Library of Medicine, which operates MEDLINEplus, and the Medical Library Association, have developed guidelines and tips for consumers to evaluate health Web site content [28]. The European Union sponsored a collaborative project called MedCERTAIN to develop a rating system to enable consumers and professionals to rate quality information on the Web. The MedCERTAIN project evolved in a project called MedCIRCLE, which has developed a metadata coding language to mark quality indicators on health Web sites [29].

## Discussion

### Search Engines and Mediators of Health Information

#### Electronic and Human Mediation

Search engines serve an essential function in enabling users to find relevant information on the Internet. Recognizing the challenges of sorting the enormous amount of information on the Internet, many organizations are augmenting or mediating the results of electronic searches. Mediation can be either electronic or human-augmented techniques for reviewing information and making a pre-selected set of information available to consumers. One challenge to search engines and human mediators is making access to personalized information as effortless as possible, as consumers rarely use even the advanced search features currently available to them [30].

#### How Search Engines Work

Search engines and Web directories play a central role in facilitating access to health information. Web directories are organized Web site listings put together by human reviewers. Search engine listings are put together by automated systems and lack a navigable structure. Directories usually concentrate on indexing Web sites, while search engines typically index individual Web pages. Consumer searches for keywords will result in a valid match only if the keyword appears in the Web site's description. Hybrid models of search engines and directories are common.

#### Search Engine Indexing and Retrieval Methods

Virtually all commercial search engines rely on large powerful databases that utilize automated search agents called robots ("bots"), crawlers, or spiders. Search agents crawl the Web continuously to index information on Web sites. Crawlers capture metadata, page titles and textual content, and add them to the search engine's index or main database. The search engine's algorithm compares indexed data to the user term to process a search. Search engine algorithms are quite complex and scientific. They make frequent use of complementary directories aimed at optimizing and positioning Web sites in the right categories. Search algorithms are closely guarded as proprietary corporate information [31].

Current metrics for evaluating search engines include initial page retrieval capacity and the ability to revisit Web sites to update information. Currency of information, as demonstrated by elimination of non-working links to Web sites is also a performance metric. These criteria are features of business performance, not necessarily the content relevance or quality of the sites returned by a search.

Content and format of Web sites determine how they are indexed by search engines. Some search engines use keyword location, frequency, phrasing, and density as indexing and ranking factors. Type and number of links associated with a Web site are common indexing factors. Web sites also use tags to identify certain types of information. Search engine databases include only Web sites that have been registered with or indexed by the search engine-hence the importance of Web site developers making their sites accessible to automated agents, or becoming known to directory developers.

#### Ranking and Ratings

Ranking of sites in the final display of search results is of great importance to Web sites, users, and search engines. Ranking effectively drives the likelihood of particular sites being recognized and visited because, as noted, consumers rarely look at more than three pages of results. A poorly designed or executed search may produce an unwieldy list of Web site results that is difficult to navigate. Alternatively, searches that are too narrowly drawn may omit important sites.

Paid preference and placement by search engines also affects which sites are retrieved in a given search [32]. A study by CWW demonstrated that consumers experience considerable confusion about paid listings, and may not distinguish them from other returned listings [33]. The Federal Trade Commission has also expressed concern about how paid placement is disclosed to consumers, and has warned search engines to clearly distinguish advertising from search returns. Search engines may operate their own paid placement programs or obtain search results from third parties, who in turn operate paid placement programs.

#### Mediated Searches

Mediated searches may be as simple as having a librarian assist with a search, or they may be based on much more complex algorithms. Participants in the URAC/CWW stakeholder group noted that medical and general librarians play an important role in helping large segments of the population retrieve online information and learn effective search strategies. More complex mediated search strategies employ both human mediation and electronic queries to interface with users and focus a search.

Many search engines offer filters that allow users to exclude unwanted search results, most typically pornographic sites. Users, including libraries, can also install blocking software to prevent unauthorized use. However, this electronic mediation may unintentionally block desired health information and create an access barrier. For example, because pornography-blocking software and filters cannot perfectly distinguish between pornographic and non-pornographic Web sites, such products may block access to legitimate health-information sites, particularly those related to sexuality [34].

Gateways employ filters, either electronic or human, to accept or reject types of sites of information based on preset criteria. Gateways are used to organize information on the Internet through selection of resources based on quality and relevance of information to a particular audience. Internet resources are reviewed, classified, and stored with descriptive information. In the US, healthfinder.gov®, is a widely used gateway to selected consumer health and human services information resources provided by US government agencies and other organizations serving the public interest [35].

Participants in the stakeholder meetings noted that domain name extensions such as .com or .org could be used as a distinguishing feature of Web sites for the purpose of focusing search efforts. The World Health Organization is considering the feasibility of requesting a "dot health (.health)" extension for a pre-selected set of trusted Web sites [36]. In informal proposals describing the .health domain name, the extension is proposed for health information, services and organizations under a framework promoting minimum standards of conduct. Oversight of Web sites would be delegated to independent verifying organizations. The advantage to sites for adhering to standards of content quality would be more ready identification of sites by search engines as a result of the .health domain name.

### Stakeholder Discussion of Literature Review

#### Research Needs to Address Consumer Evaluation of Web Quality

There is great variation in how consumers seek information via the Internet, and in how successful they are in searching for health information. Since there is significant consumer-level variation in how consumers search for health information, search algorithms that support variation and still return expected results will meet consumer needs most effectively. Additional research is needed on information needs of different consumer segments and how to effectively educate differing consumer segments to improve the results of their health searches. Research is needed on how to efficiently validate the quality of Web sites and communicate this information to consumers.

#### Research Needs for Web Site Quality Indicators

There is a need for tools to enhance recognition of quality Web sites by consumers and search engines. Such tools may be implemented by Web sites themselves, for example through increasingly sophisticated coding to highlight quality indicators. The MedCERTAIN project has been created precisely to address this issue, and has developed the HIDDEL vocabulary to mark features of Web sites [29]. Technical tools can be used to direct consumers more effectively to relevant, high quality information. In addition, since there are currently multiple tools for either self-evaluation or third party evaluation of Web sites, future research should be undertaken to validate these tools.

As noted, gateways filter information to increase its relevance to consumers and provide expert assessment regarding validity of sources is available. It may also be useful to develop more sophisticated search models for providing useful and relevant information to consumers via customization approaches. Such approaches could potentially be embedded in search algorithms. In addition, more research is needed on the impact of Internet-based health information on outcomes. The benefits and risks of health information, both from a health outcome and a system outcome (quality, cost), are poorly understood and should be examined further.

#### Research Needs for Search Factors Influencing Search Results

Search engines are increasingly important as a tool for locating and organizing information from the vast Internet resource. The volume of information on the Web is so significant that consumers may need different types of mediators, such as search engines or librarians, to help manage the volume of information. Human assistance is also helpful to counteract electronic spoofing and to help consumers overcome limitations in their search strategies.

To effectively improve health searches, more information is needed about search algorithms and how quality factors are identified in the algorithms. Search engines are also developing technology to search for synonyms, which may enhance health searches conducted by laypersons. It may also be helpful for search engines to develop methods to distinguish health related searches from other types of searches, rather than using a simple word match. Search technology to intuit consumer needs more effectively and learn from repeated searches could help search engines steer consumers to quality results. New technologies may ultimately be more effective than electronic filtering, requiring consumers to apply filters, or modifying their search strategies.

With technology advances, search engines may be able to identify quality proxies that could improve page rankings of high-quality Web sites. Search engines could, for example, give higher ranking to "official sites" for diseases. They could also piggyback onto credibility assessments provided by groups such as healthfinder.gov, or give higher ranking to sites listed in directories from trusted independent sources. Ultimately, adoption of technological solutions depends on the ability of researchers to understand the relationship between electronic proxies for quality and actual quality of content.

### Discussion of Stakeholder Recommendations for Next Steps

The URAC and CWW expert panels discussed consumer, Web site, and search engine factors that influence the outcomes of health searches. In the course of discussion, they developed a number of recommendations for future research and development (Textbox 1). Their recommendations fell into several categories: needs for health services research, consumer and provider education, technological improvements, and development of tools and information to improve the results of health searches. For some recommendations, the evidence base for implementation is strong; for others, not. Implementation of some recommendations will be enhanced by creation of a national research agenda for health information and targeted funding to study and improve consumers' ability to locate and retrieve quality health information on the Internet. Other recommendations could be embraced at any time by researchers, educators or technology organizations as a business need becomes increasingly evident.

**Recommendations of the Group**

***Leadership for Health Search Improvement***

- Organizations concerned about the quality and accessibility of health information online should continue to collaborate to promote "health search literacy."
- Collaborators should convene a leadership summit on health search literacy to discuss feasibility and implementation of many of the recommendations herein.
- Collaborating organizations should
- work with funding organizations to develop a comprehensive long-term research agenda to improve health searches and increase access to quality health information;
- develop enhanced research methodologies to evaluate the quality, impact, and effectiveness of online health information.

***Consumer-directed Tools***

- Create tools to support consumer health-information needs, including preset, prescreened health bookmarks and more guidance on how to reach health gateways and portals containing trusted health content.
- Develop and circulate a public domain brochure on health search strategies that could be branded and distributed by physicians, employers, health plans, and others to educate consumers.
- Develop public domain interactive, validated search strategy content pages that could be branded and used by health Web sites.

***Research Needs***

- Identify the search needs and capabilities of diverse populations of searchers, including culturally diverse users and searchers with health needs of differing intensity and severity.
- Develop more understanding about how consumers interpret online health information, assess its credibility, and make health-related decisions.
- Research the relationship between consumer search strategies and consumer expectations for results to determine effective approaches for conveying information on the Internet.
- Research factors affecting physician assessments of Web-based information and how quality content affects physician recommendations to patients about online health-information resources.
- Assess the relationship between expert accreditation, quality seals, ratings and content quality, as well as the impact of such endorsements on both consumer behavior and Web site behavior.
- Research the correlation between Web site traffic volume and consumer satisfaction, particularly for health Web sites where there is variation in dimensions of quality such as accuracy, comprehensiveness, ease of navigation, and reading level.
- Evaluate content quality of Web sites in different domains, (eg, .gov, .edu, .com, and .org) to identify similarities and differences related to quality within and across categories of Internet domain names
- Evaluate the impact of Internet-based health information on health outcomes: utilization, behavior change, knowledge, burden of illness and disease, or other measures.
- Research the relative effect of each component of a search algorithm (word frequency and placement, links, etc) for finding health information.
- Validate elements of some search algorithms, such as link frequency, as indicators of value/quality.
- Conduct periodic studies to monitor changes in accuracy and quality of content over time, including updating findings from the California HealthCare Foundation /RAND study [5].

***Education Agenda***

- Develop models for offering health search education at teachable moments and in diverse consumer settings.
- Promote dissemination of existing educational tools and resources to assist consumers in evaluating health information on the Web more effectively.
- Develop user-appropriate tools and approaches to assist Internet users with special needs. High priority user groups may include disability, low literacy, and non-English speaking groups.
- Urge provider organizations to educate provider members on the value of offering Internet information and interactive learning recommendations as part of the therapeutic intervention.
- Educate health Web site developers on how to make information easy to find and how to meet the content-level of their intended users.
- Urge education organizations, in collaboration with health organizations, to develop a school-based or publicly available health search curriculum.

***Technology Improvement Agenda***

- Continue to develop interactive features on search engines and sites to customize and personalize health searches.
- Develop more functionality for search engines to enhance selected health queries by offering additional relevant information.
- Develop technological markers or indicators that could be uniformly applied by Web site developers to indicate accuracy and comprehensiveness of health Web sites.
- Develop codes to indicate when information on a Web site supercedes previous information.
- Develop collaborations between health quality and search engines experts to develop codes for validated quality proxies.
- Develop search technology similar to that used in the commercial sector to direct consumers to related, relevant information based on both searching and viewing behaviors.
- Enhance personalized searches by building search engine capability to "learn" from repeated searches and user behavior.

***Expanding the Market for Quality***

- Develop a health equivalent of "BizRate" or "eBay" surveys that can be used by consumers to evaluate Web sites after viewing. Existing models for such a survey could be adapted and disseminated.
- Sponsor a competition for individuals or organizations to design a search algorithm that returns the most credible health results as evaluated by experts. Design a separate contest for the most effective business plan to make the business case for building quality factors into health searches.

## Conclusion

The Internet has opened a vast library of information to consumers of health information and made that information more accessible than ever before. This represents a significant step forward for consumers. However, the volume of information and the variable quality of information has created new interpretive challenges. Now, one great challenge is helping consumers find the information they want that is also accurate, reliable, and presented in an accessible format. Searches for health information rely on a complex interplay of search algorithms, Web site content and coding, and consumer behaviors. The recommendations presented here address each of those factors with ideas for further research as well as more immediate recommendations for action. This agenda is a start at maximizing the potential of the Internet to deliver high-quality health information for diverse users.

## Appendix 1

### URAC/CWW Stakeholder Summit Participants (titles as they were at time of the summit)

Lois C Ambash, PhD, MA, MS, President, Metaforix Incorporated; David R Baker, Senior Publishing Advisor (Internet), ODPHP Office of Public Health and Science; Cynthia Baur, PhD, Health Communication and e-Health-Advisor, DHHS ODPHP; Beau Brendler, Director, Consumer WebWatch.org, Consumers Union; Patrick E Cochran, PhD, CSD, Periodic Paralysis Association; Stephen J Downs, SM, Senior Program Officer, The Robert Wood Johnson Foundation; Mary P Doyle, MD, Associate Medical Director, Anthem Blue Cross & Blue Shield; Joan H Dzenowagis, PhD Project Manager, Health InterNetwork, World Health Organization; Kathleen C Eckett, MHA, RN, CPHQ, Director, Quality Management, Group Health Incorporated; Tom Eng, MD, President, EvaluMetrix LLC & eHealth Institute; Ray Fabius, MD Global Medical Leader, General Electric; Steven Figman, Director, eBusiness Strategy, Johnson & Johnson-Ortho Biotech; Susannah Fox, Director of Research, Pew Internet & American Life Project; Alan Greene, MD, President of High Ethics, founder www.drgreene.com; Jennifer Haslip, Director of Communications, National Health Council;; Nancy Hitzschke, Project Manager, BlueWeb and Plan Education, Blue Cross Blue Shield Association; Leslie Hsu, Chair, healthfinder.gov Steering Committee, DHHS ODPHP; Ann Mond Johnson, President, Subimo, LLC; Kevin Kujawa, CIO, American Specialty Health; Adria Kyne, Manager, Search Engine Marketing, Catalyst on-line; Devin A Jopp, Chief Information Officer; Health Insurance Association of America; Eve-Marie Lacroix, MS, Chief Information Officer, Public Services Division; National Library of Medicine; Daniel J Lorence, PhD, JD, Pennsylvania State University, School of Information Sciences and Technology; Sarah Loughran, MBA, MHSA, Senior Vice President, Health Grades, Inc; Scott McWilliams, Chief Technology Officer, VitalSeek / Today Communications, Inc.,; Janice M Morton, MD, Schering-Plough, Internet Strategy; Linda Neuhauser, DrPh, Clinical Professor, School of Public Health University of California; Hana Paek, Research Associate, Health Improvement Institute; Andrew Robinson, Executive Director, Patient2Patient; Michael Patrick Rutter, BA, Senior Project Manager, Harvard Medical School Harvard Health Publications; Ronni Sandroff, Director of Health Information / Health Editor, Consumer Reports, Consumer Reports on Health; Skye Schulte, MS, MPH Content Editor / Marketing Liason, HealthGate Data; Joshua Seidman, PhD, Executive Director, Center for Information Therapy; Al Shar, Vice President, Information Technology, Robert Wood Johnson Foundation; Gail Shearer, Director of Health Policy Analysis, Consumers Union; Michele Spatz, MS, Director, Planetree Health Resource Center; Mark Spranca, PhD, Director, Center for Healthcare and the Internet, RAND; Beverly Thomas, JD, Senior Attorney, Federal Trade Commission; James "JT" Thome, Vice President, Business Operations, WebMD

***URAC Staff***

Guy D'Andrea, Senior Vice President, URAC; Lisa A Gallagher, Senior Vice President, Information & Technology Accreditation, URAC; Liza Greenberg, RN, MPH, Vice President, Research and Quality Initiatives; Ryan F Lawton, MS, Director, Information & Technology Accreditation, URAC

## Multimedia Appendix

Powerpoint Presentation from URAC about the Project

## Abbreviations

CWW: Consumer Web Watch
RAND: Research and Development Corporation
URAC: Utilization Review Accreditation Commission
