# Supplementary material for: Setting the Public Agenda for Online Health Search: A White Paper and Action Agenda
Source: J Med Internet Res. 2004 Jun 8;6(2):e18. doi: 10.2196/jmir.6.2.e18 (PMC1550592; doi:10.2196/jmir.6.2.e18)
Supplement: Supplementary file 1 [file jmir_v6i2e18_app2.ppt]

## Slide 1
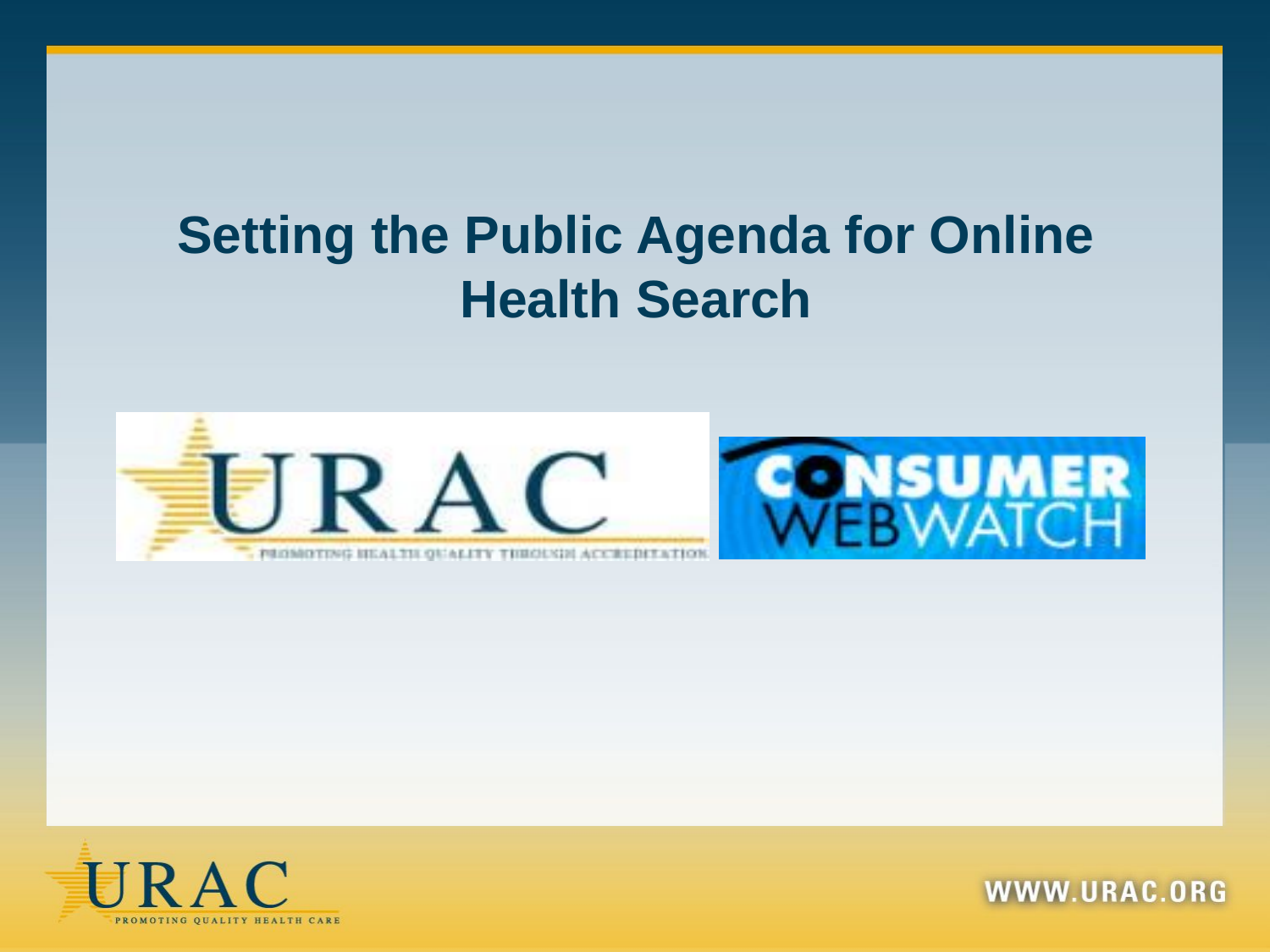

# Setting the Public Agenda for Online Health Search

## Slide 2
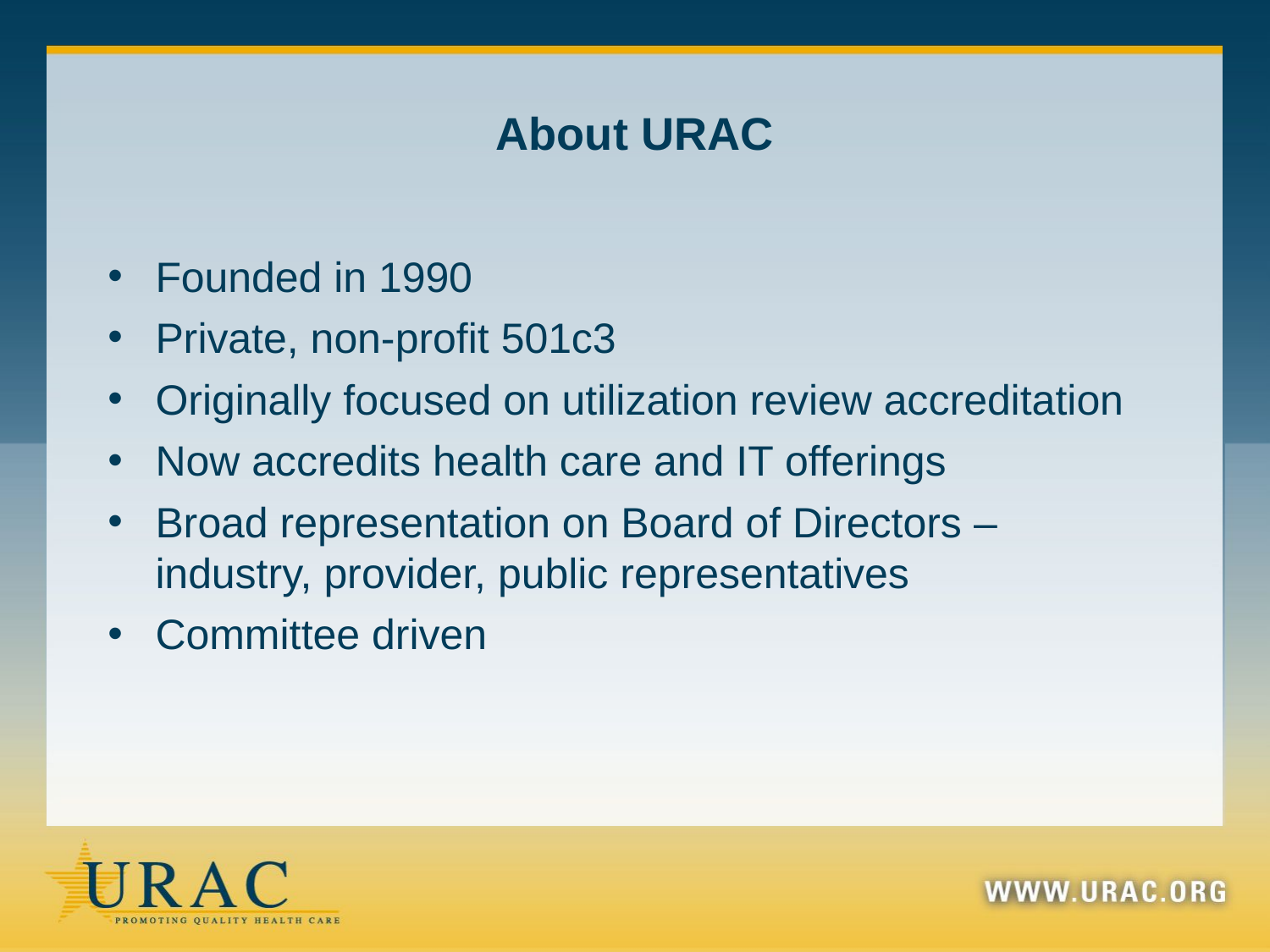

# About URAC
Founded in 1990
Private, non-profit 501c3
Originally focused on utilization review accreditation
Now accredits health care and IT offerings
Broad representation on Board of Directors – industry, provider, public representatives
Committee driven

## Slide 3
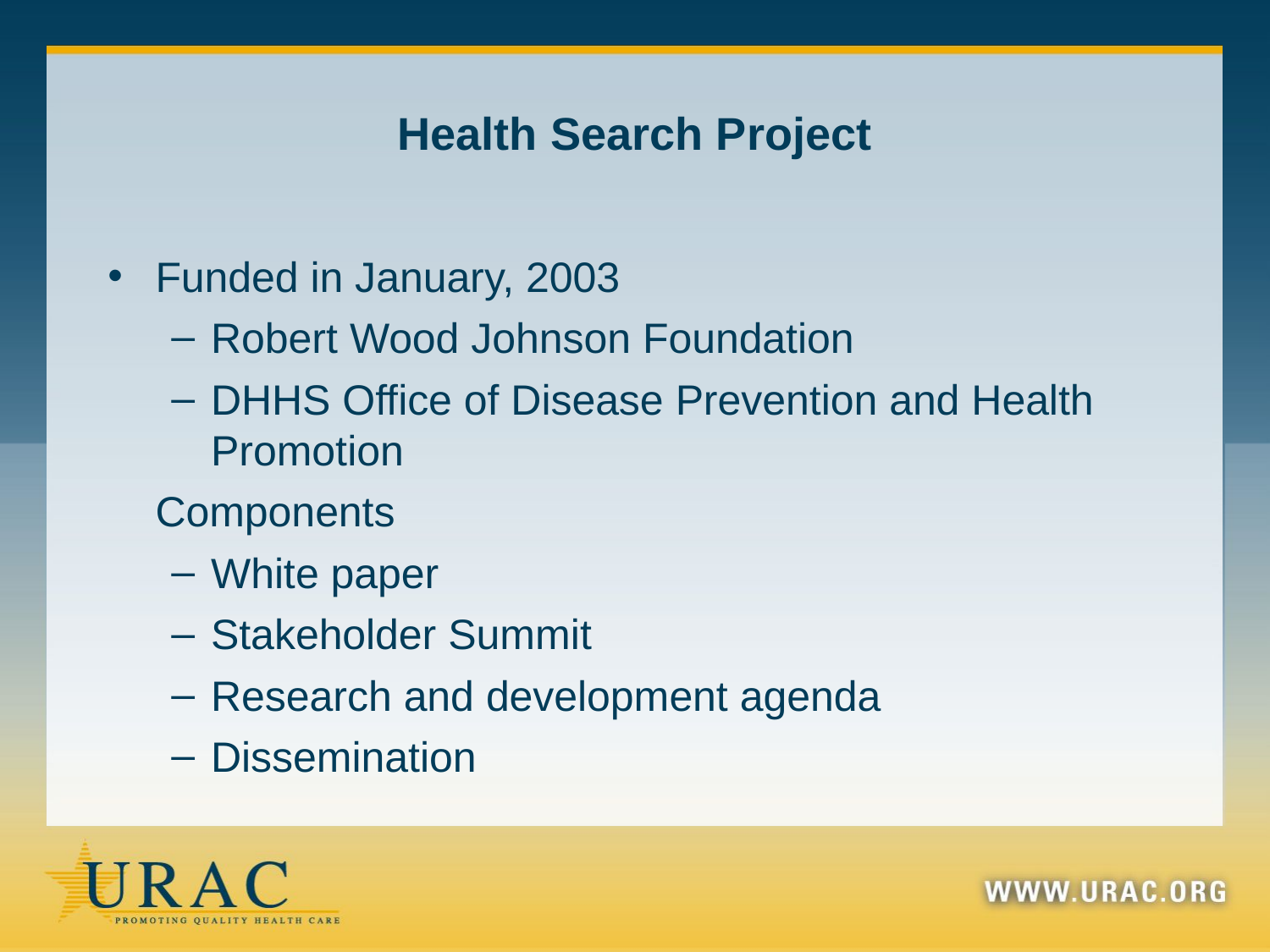

# Health Search Project
Funded in January, 2003
Robert Wood Johnson Foundation
DHHS Office of Disease Prevention and Health Promotion
Components
White paper
Stakeholder Summit
Research and development agenda
Dissemination

## Slide 4
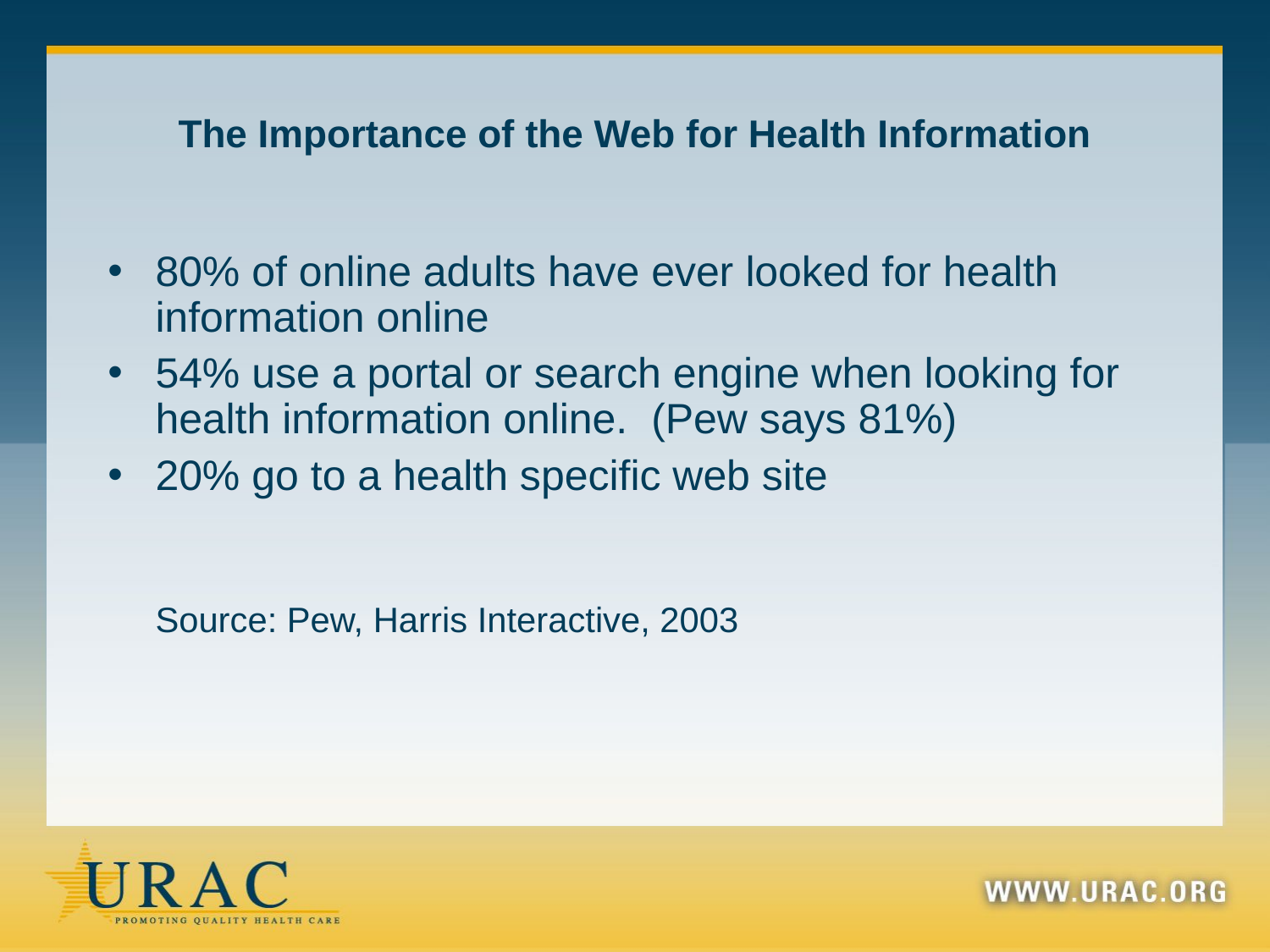

# The Importance of the Web for Health Information
80% of online adults have ever looked for health information online
54% use a portal or search engine when looking for health information online. (Pew says 81%)
20% go to a health specific web site
Source: Pew, Harris Interactive, 2003

## Slide 5
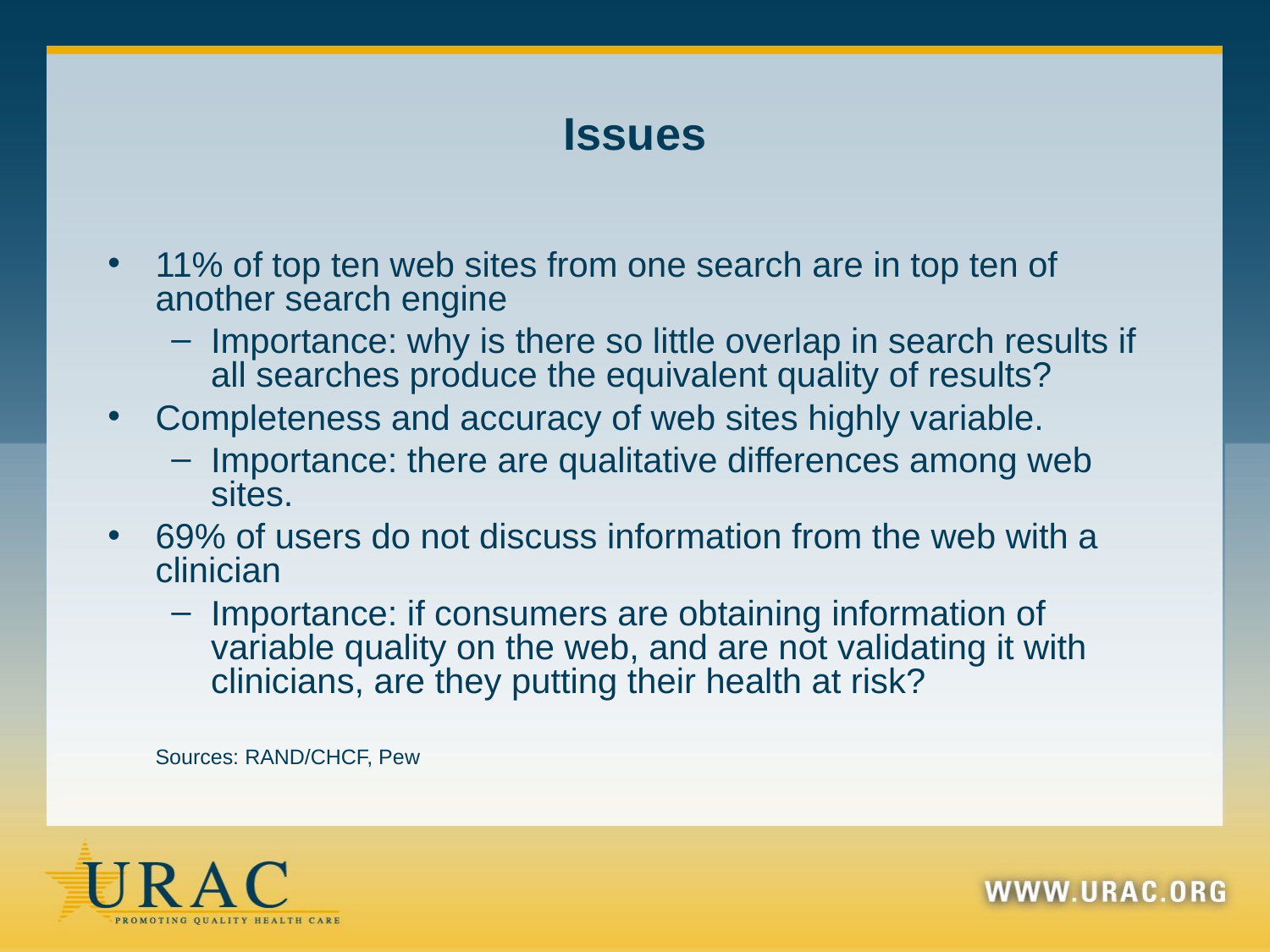

# Issues
11% of top ten web sites from one search are in top ten of another search engine
Importance: why is there so little overlap in search results if all searches produce the equivalent quality of results?
Completeness and accuracy of web sites highly variable.
Importance: there are qualitative differences among web sites.
69% of users do not discuss information from the web with a clinician
Importance: if consumers are obtaining information of variable quality on the web, and are not validating it with clinicians, are they putting their health at risk?
Sources: RAND/CHCF, Pew

## Slide 6
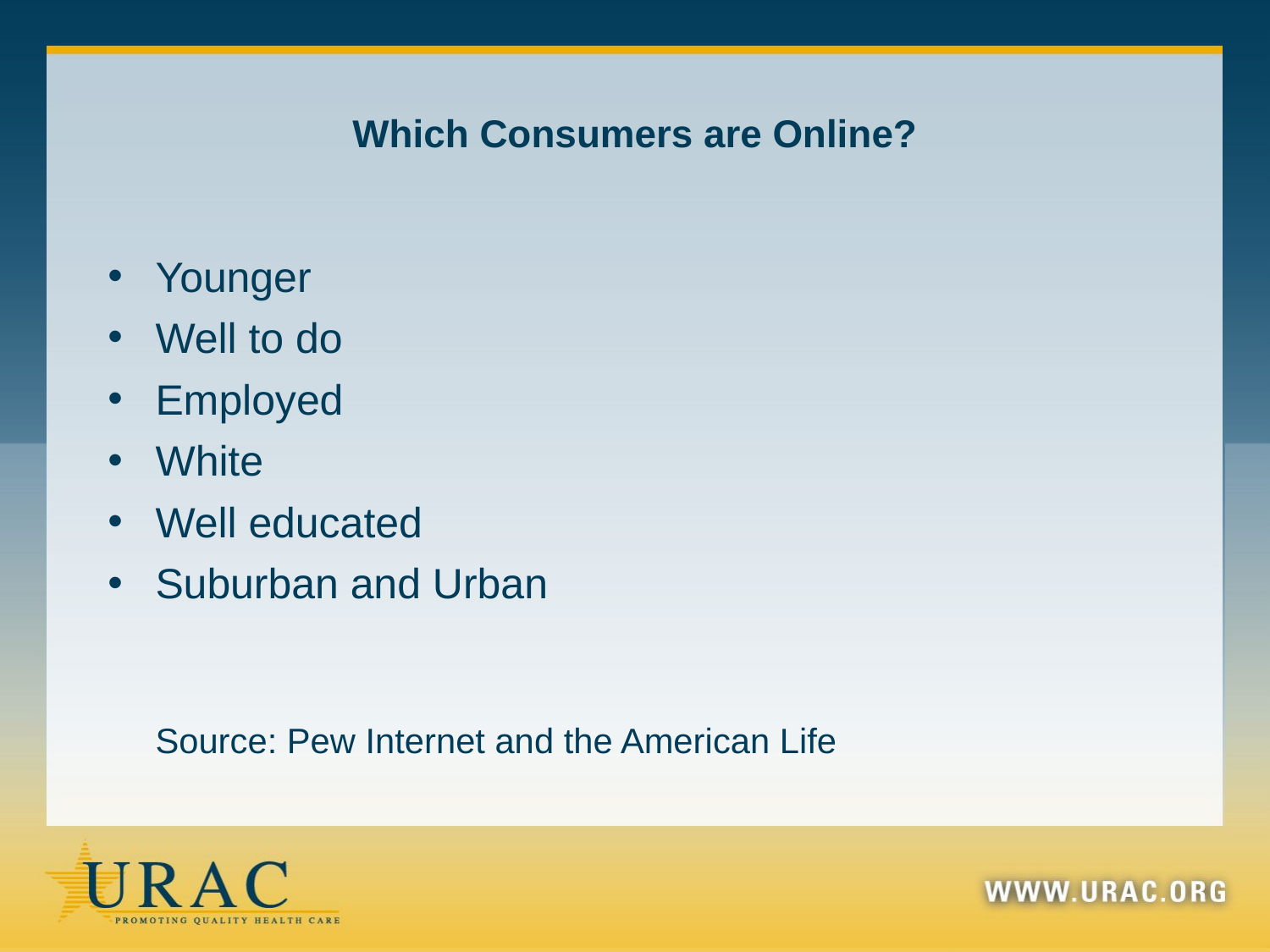

# Which Consumers are Online?
Younger
Well to do
Employed
White
Well educated
Suburban and Urban
Source: Pew Internet and the American Life

## Slide 7
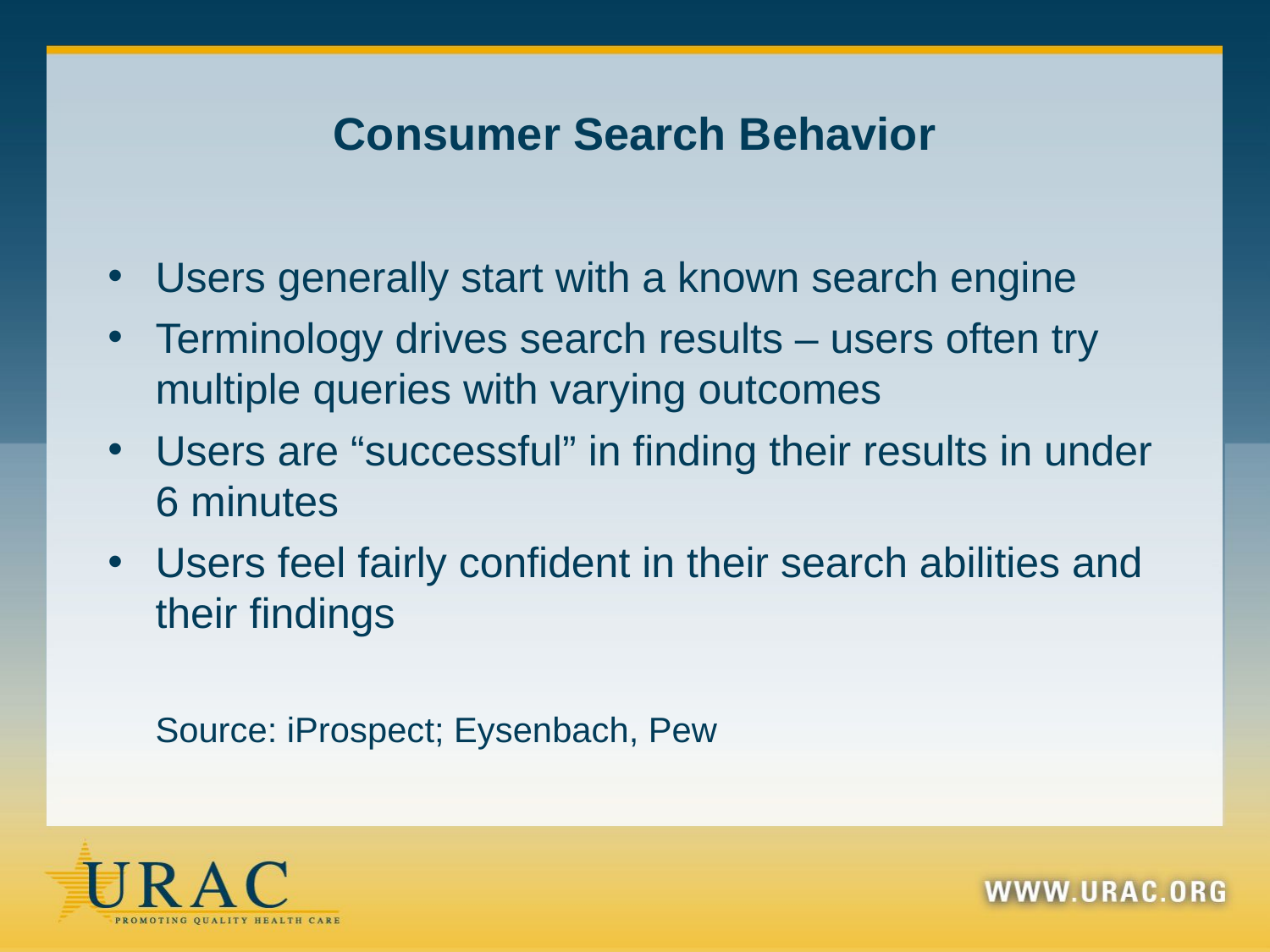

# Consumer Search Behavior
Users generally start with a known search engine
Terminology drives search results – users often try multiple queries with varying outcomes
Users are “successful” in finding their results in under 6 minutes
Users feel fairly confident in their search abilities and their findings
Source: iProspect; Eysenbach, Pew

## Slide 8
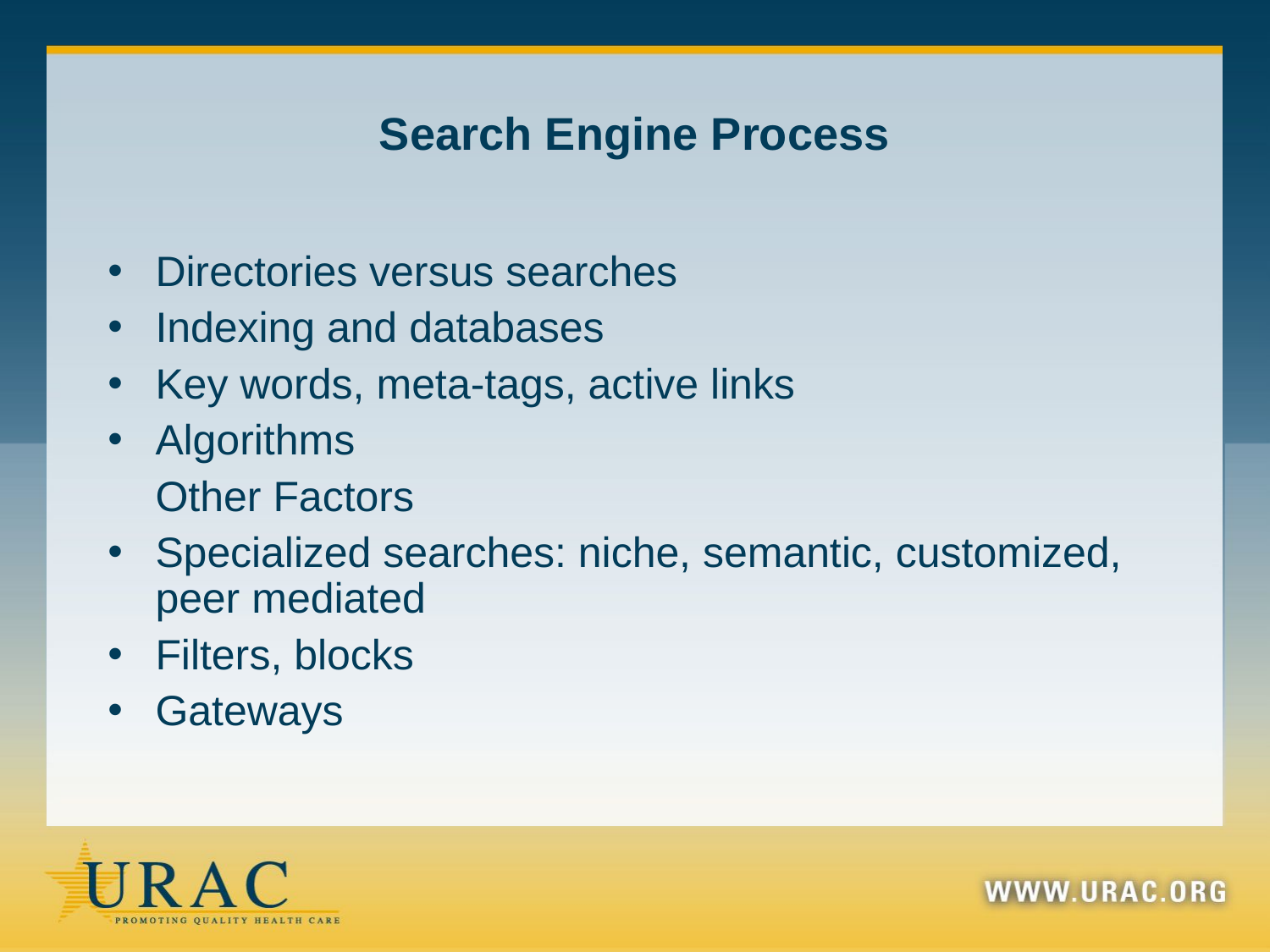

# Search Engine Process
Directories versus searches
Indexing and databases
Key words, meta-tags, active links
Algorithms
Other Factors
Specialized searches: niche, semantic, customized, peer mediated
Filters, blocks
Gateways

## Slide 9
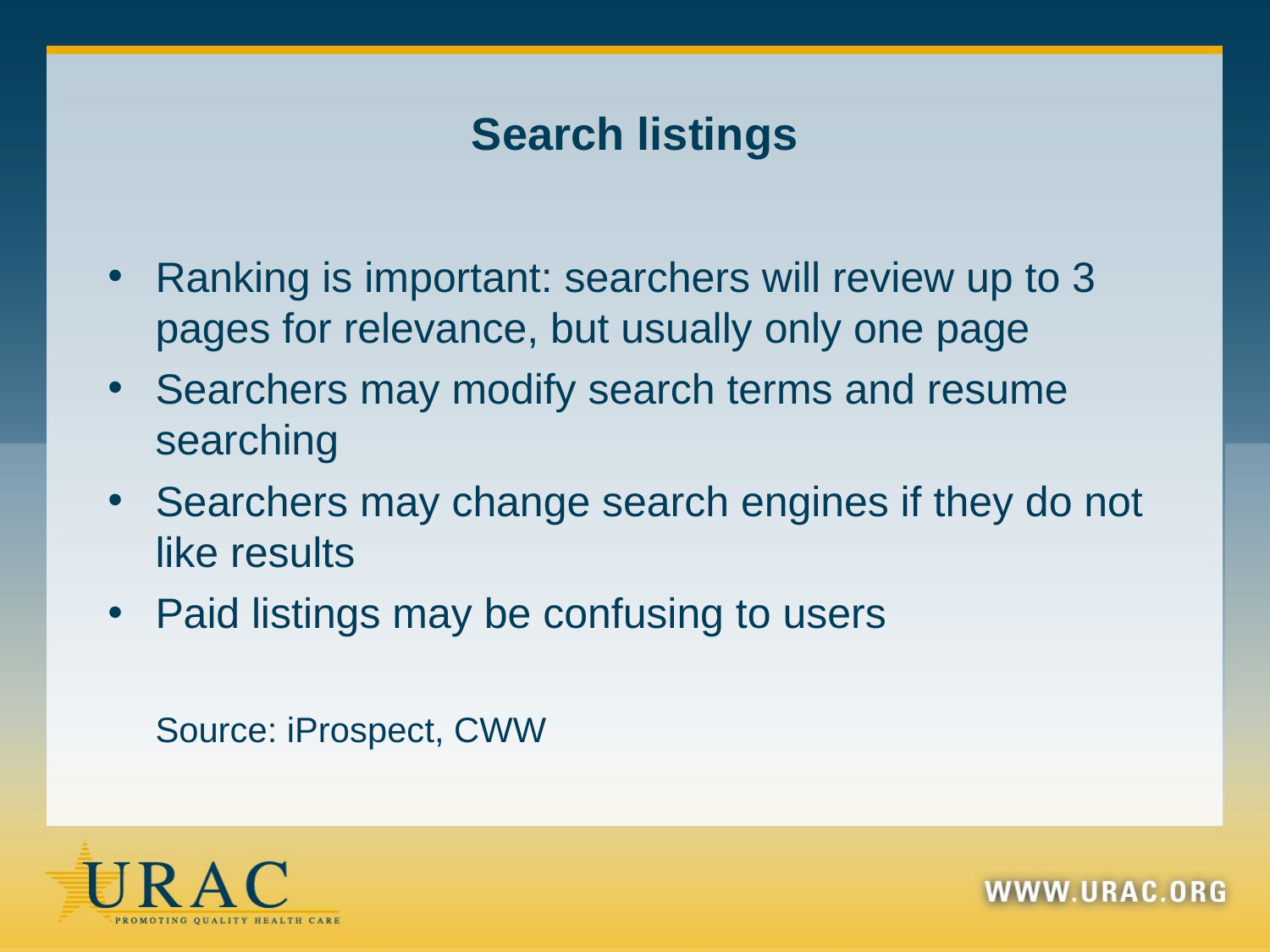

# Search listings
Ranking is important: searchers will review up to 3 pages for relevance, but usually only one page
Searchers may modify search terms and resume searching
Searchers may change search engines if they do not like results
Paid listings may be confusing to users
Source: iProspect, CWW

## Slide 10
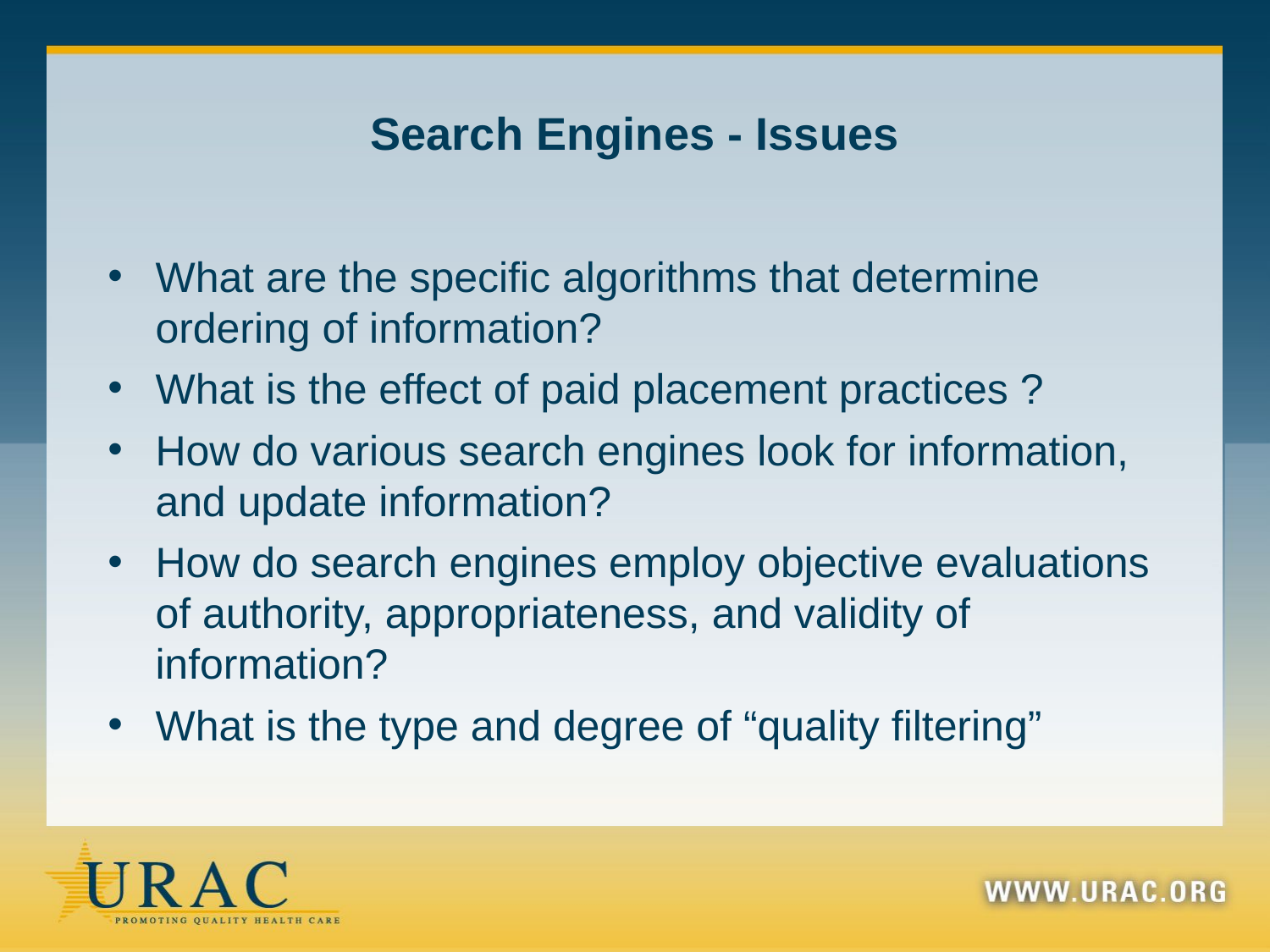

# Search Engines - Issues
What are the specific algorithms that determine ordering of information?
What is the effect of paid placement practices ?
How do various search engines look for information, and update information?
How do search engines employ objective evaluations of authority, appropriateness, and validity of information?
What is the type and degree of “quality filtering”

## Slide 11
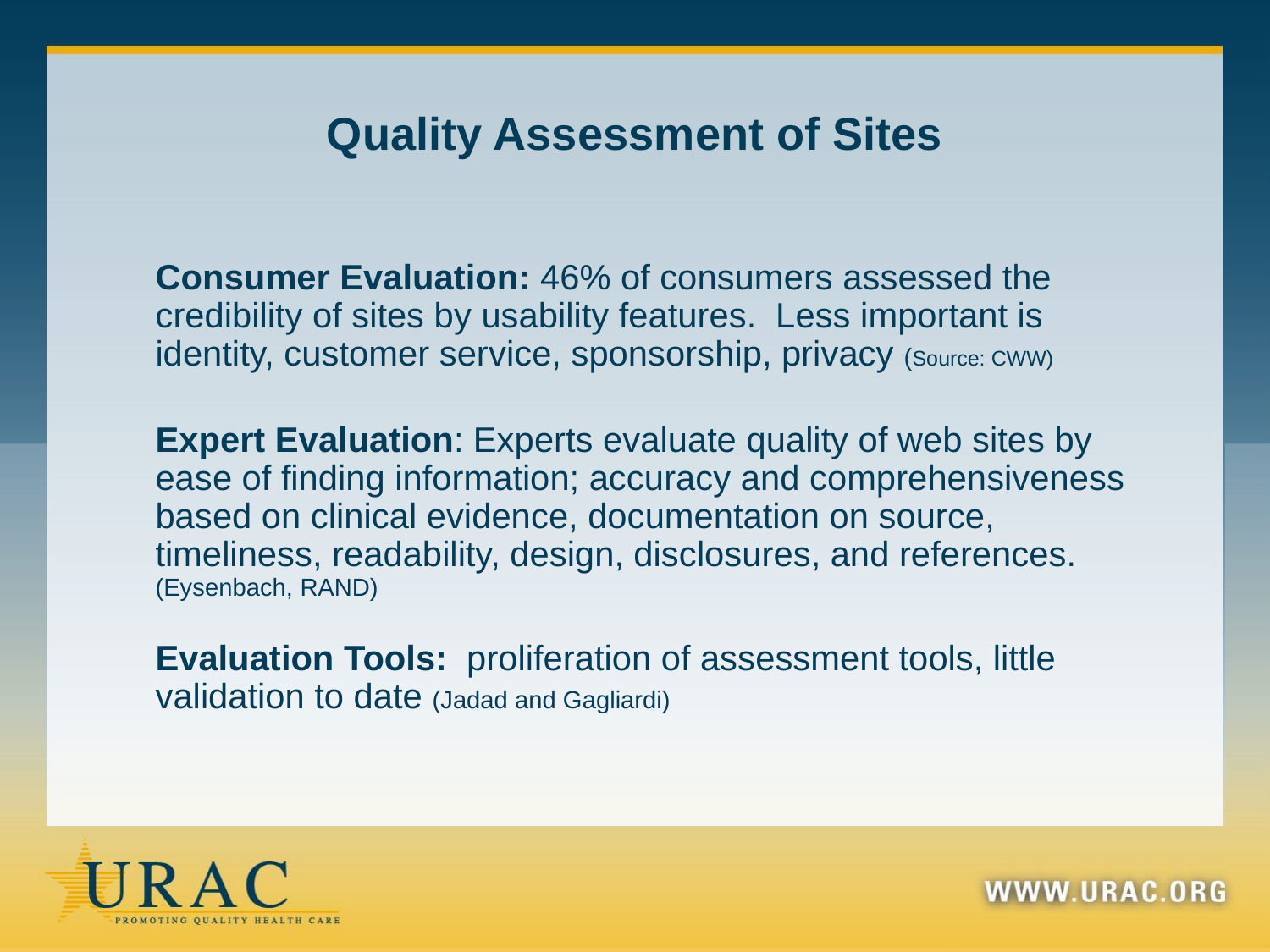

# Quality Assessment of Sites
Consumer Evaluation: 46% of consumers assessed the credibility of sites by usability features. Less important is identity, customer service, sponsorship, privacy (Source: CWW)
Expert Evaluation: Experts evaluate quality of web sites by ease of finding information; accuracy and comprehensiveness based on clinical evidence, documentation on source, timeliness, readability, design, disclosures, and references. (Eysenbach, RAND)
Evaluation Tools: proliferation of assessment tools, little validation to date (Jadad and Gagliardi)

## Slide 12
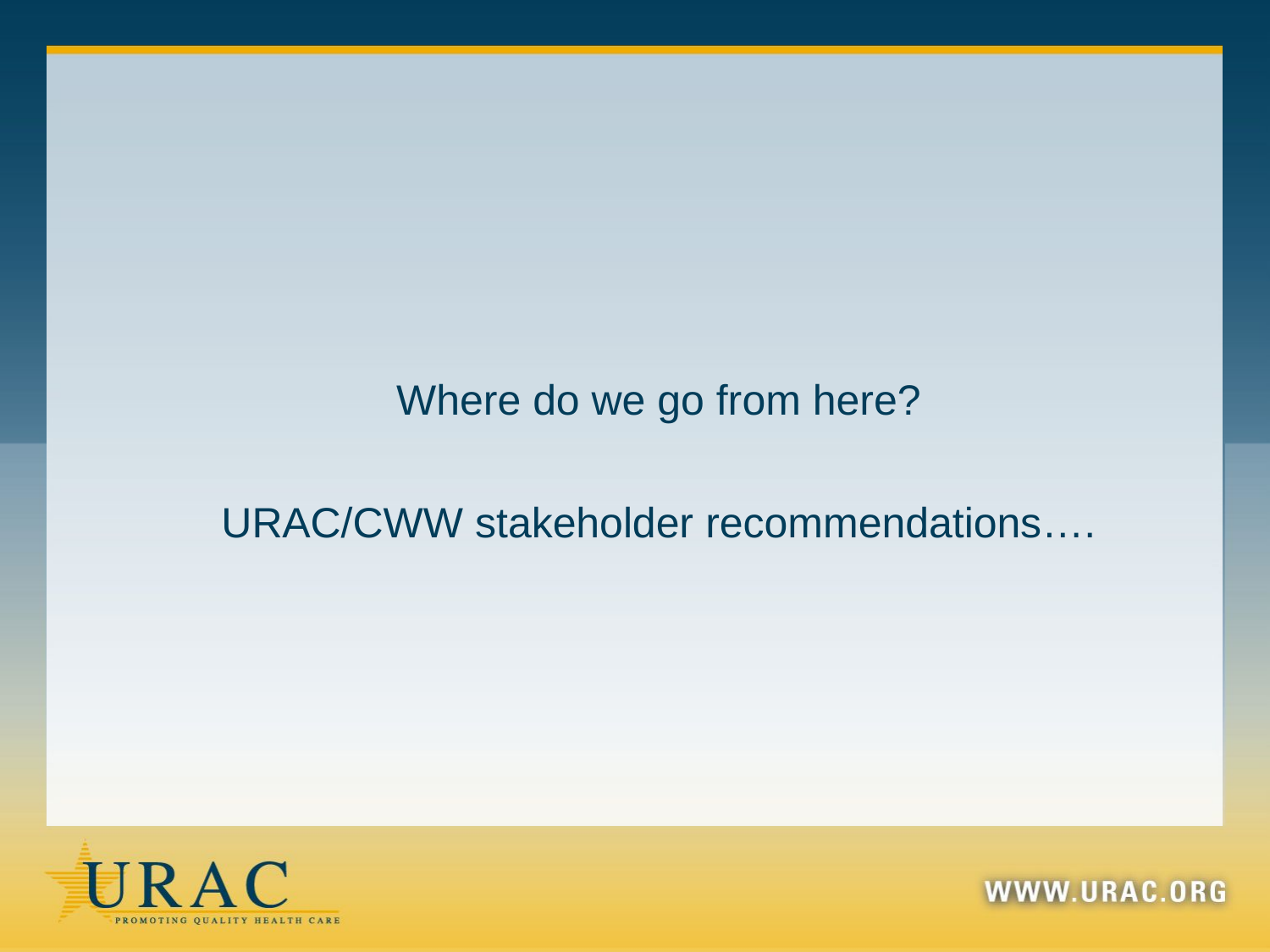

#
Where do we go from here?
URAC/CWW stakeholder recommendations….

## Slide 13
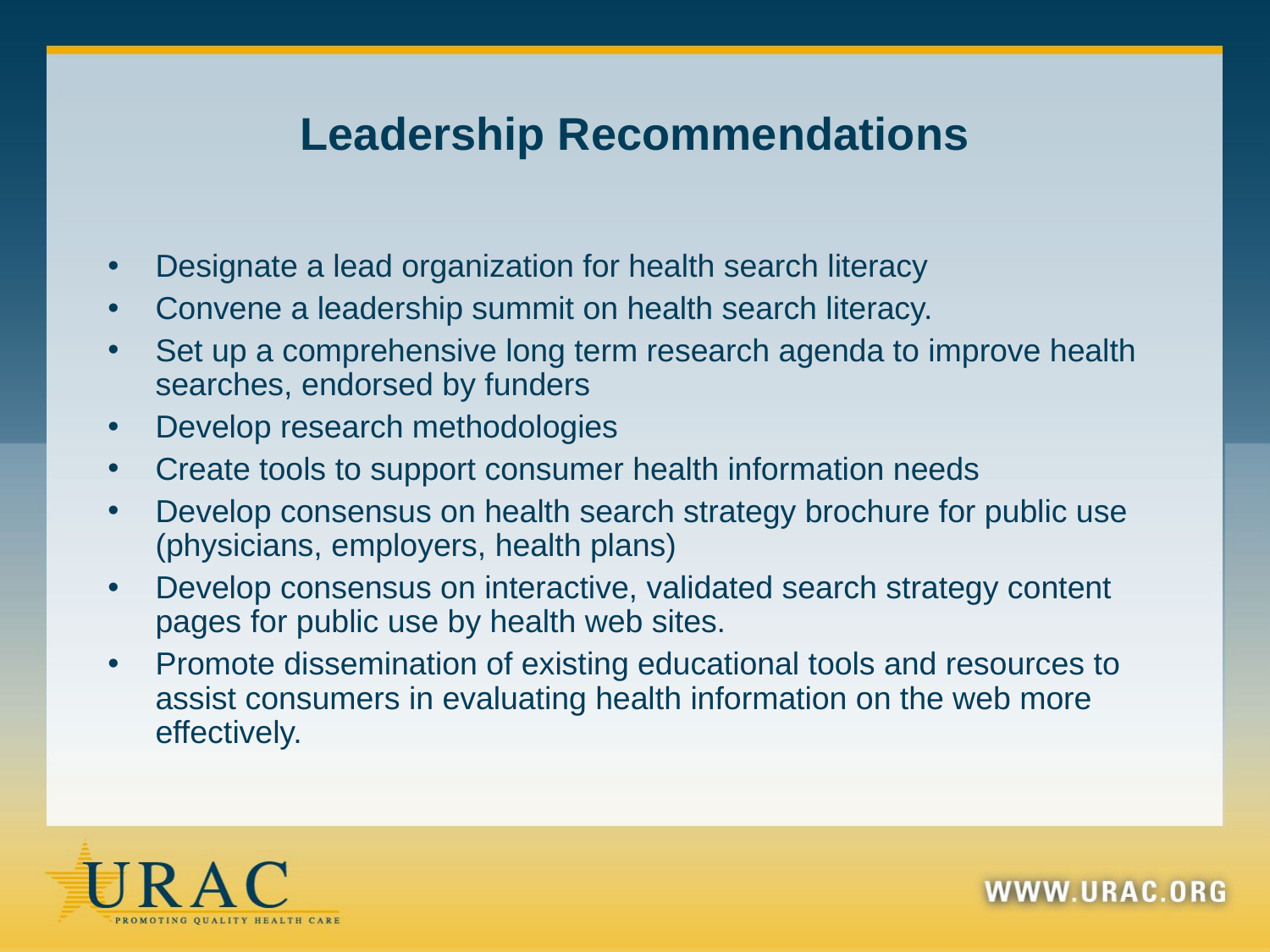

# Leadership Recommendations
Designate a lead organization for health search literacy
Convene a leadership summit on health search literacy.
Set up a comprehensive long term research agenda to improve health searches, endorsed by funders
Develop research methodologies
Create tools to support consumer health information needs
Develop consensus on health search strategy brochure for public use (physicians, employers, health plans)
Develop consensus on interactive, validated search strategy content pages for public use by health web sites.
Promote dissemination of existing educational tools and resources to assist consumers in evaluating health information on the web more effectively.

## Slide 14
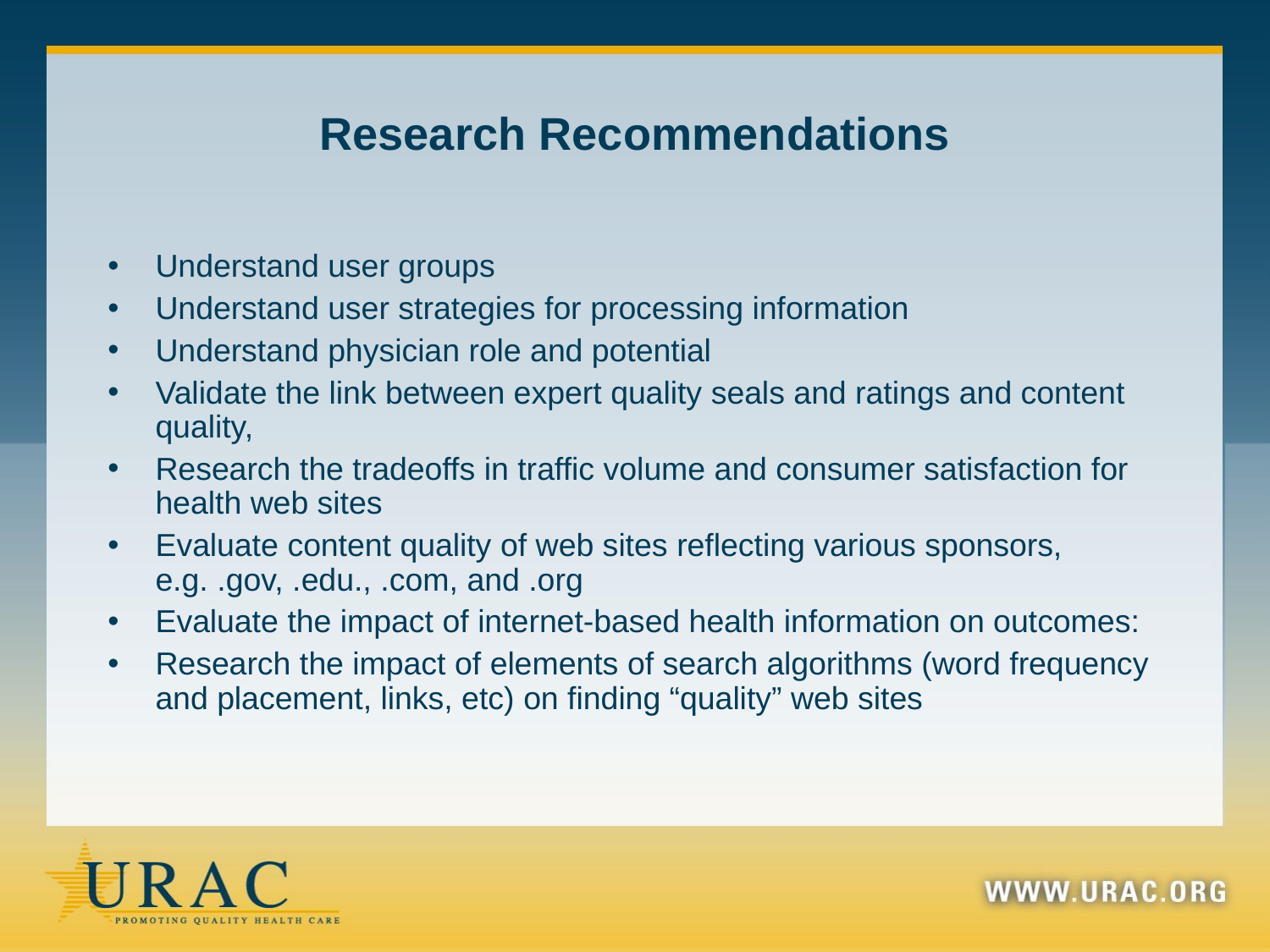

# Research Recommendations
Understand user groups
Understand user strategies for processing information
Understand physician role and potential
Validate the link between expert quality seals and ratings and content quality,
Research the tradeoffs in traffic volume and consumer satisfaction for health web sites
Evaluate content quality of web sites reflecting various sponsors, e.g. .gov, .edu., .com, and .org
Evaluate the impact of internet-based health information on outcomes:
Research the impact of elements of search algorithms (word frequency and placement, links, etc) on finding “quality” web sites

## Slide 15
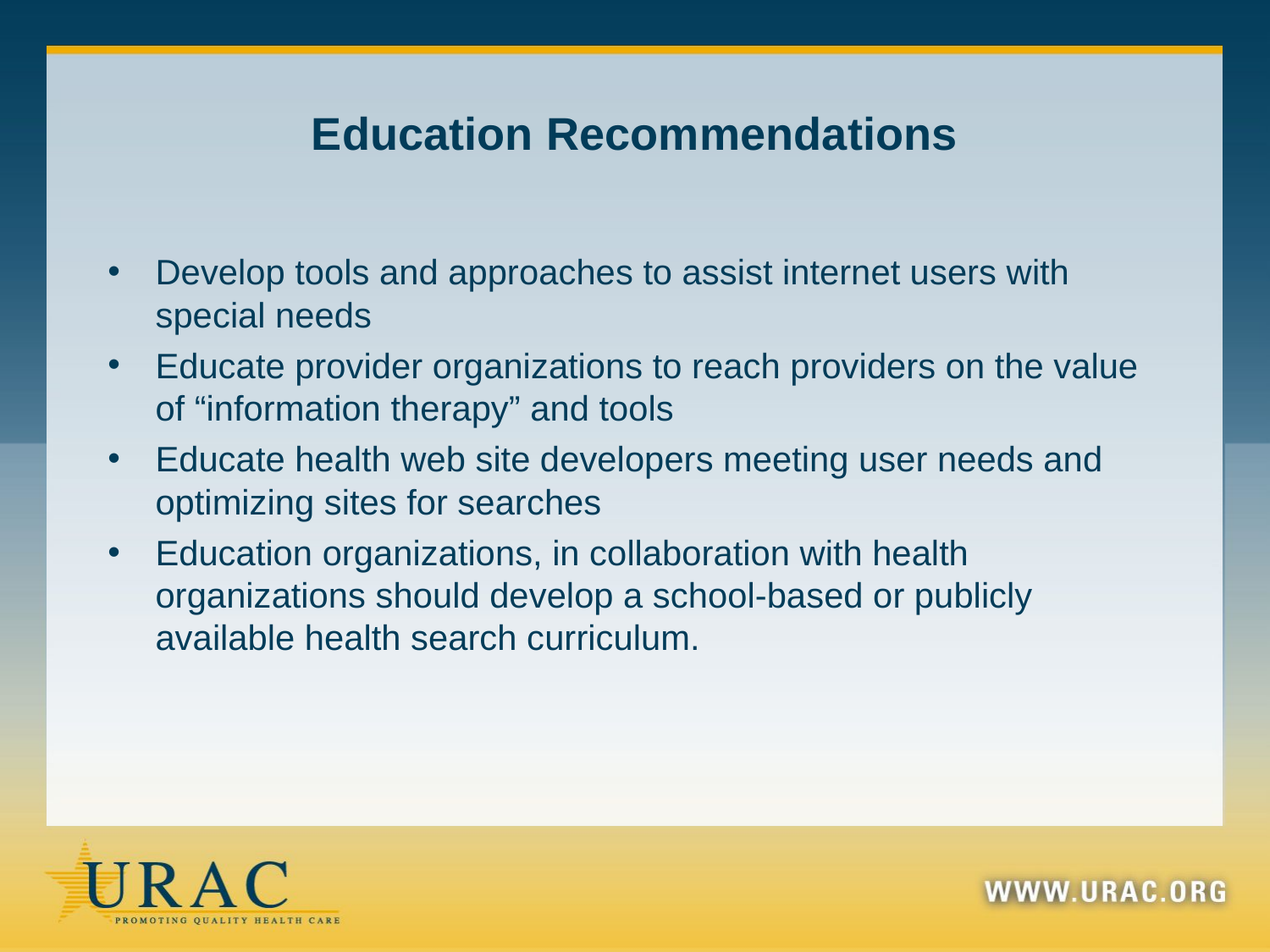

# Education Recommendations
Develop tools and approaches to assist internet users with special needs
Educate provider organizations to reach providers on the value of “information therapy” and tools
Educate health web site developers meeting user needs and optimizing sites for searches
Education organizations, in collaboration with health organizations should develop a school-based or publicly available health search curriculum.

## Slide 16
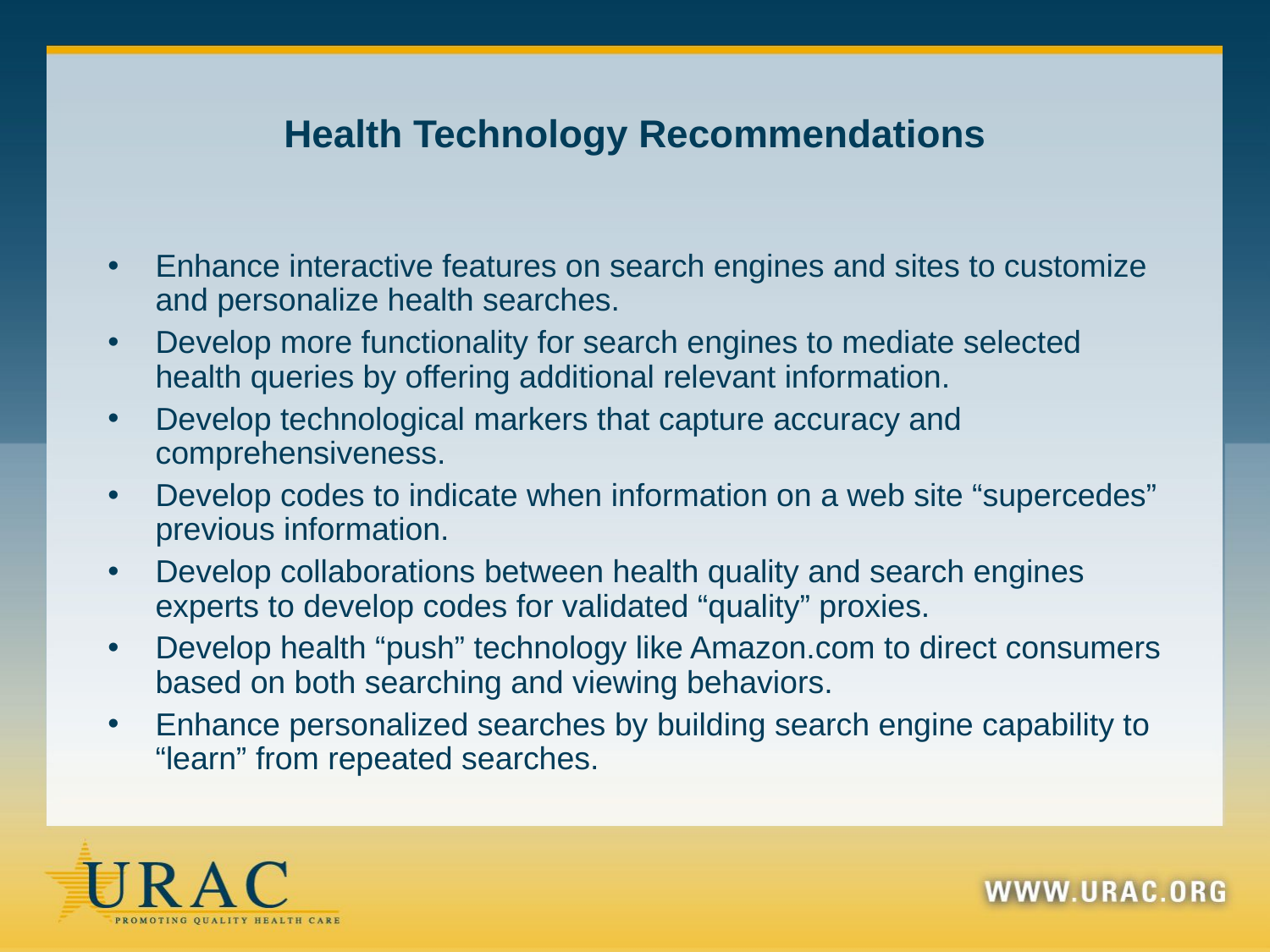

# Health Technology Recommendations
Enhance interactive features on search engines and sites to customize and personalize health searches.
Develop more functionality for search engines to mediate selected health queries by offering additional relevant information.
Develop technological markers that capture accuracy and comprehensiveness.
Develop codes to indicate when information on a web site “supercedes” previous information.
Develop collaborations between health quality and search engines experts to develop codes for validated “quality” proxies.
Develop health “push” technology like Amazon.com to direct consumers based on both searching and viewing behaviors.
Enhance personalized searches by building search engine capability to “learn” from repeated searches.

## Slide 17
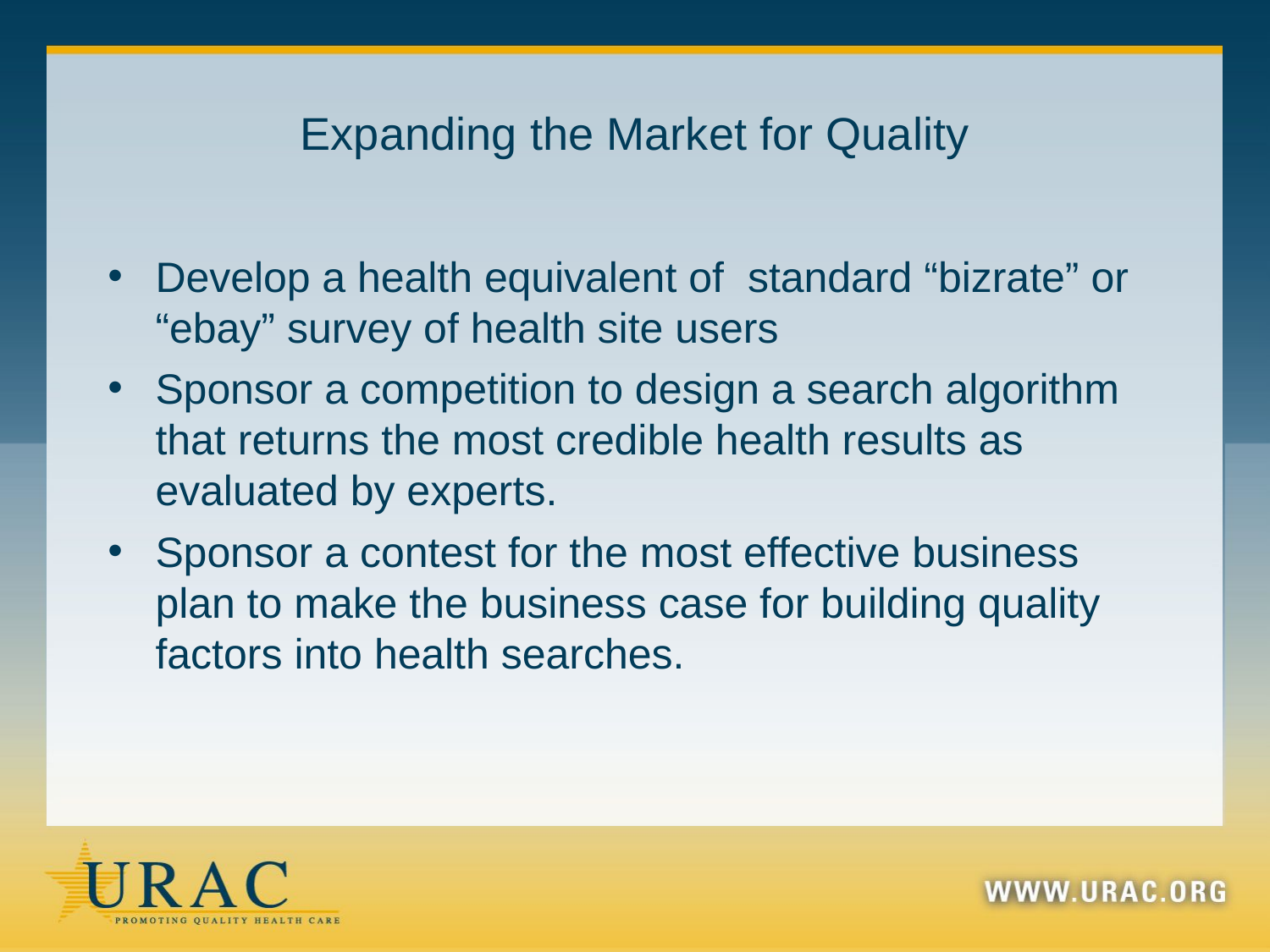

# Expanding the Market for Quality
Develop a health equivalent of standard “bizrate” or “ebay” survey of health site users
Sponsor a competition to design a search algorithm that returns the most credible health results as evaluated by experts.
Sponsor a contest for the most effective business plan to make the business case for building quality factors into health searches.

## Slide 18
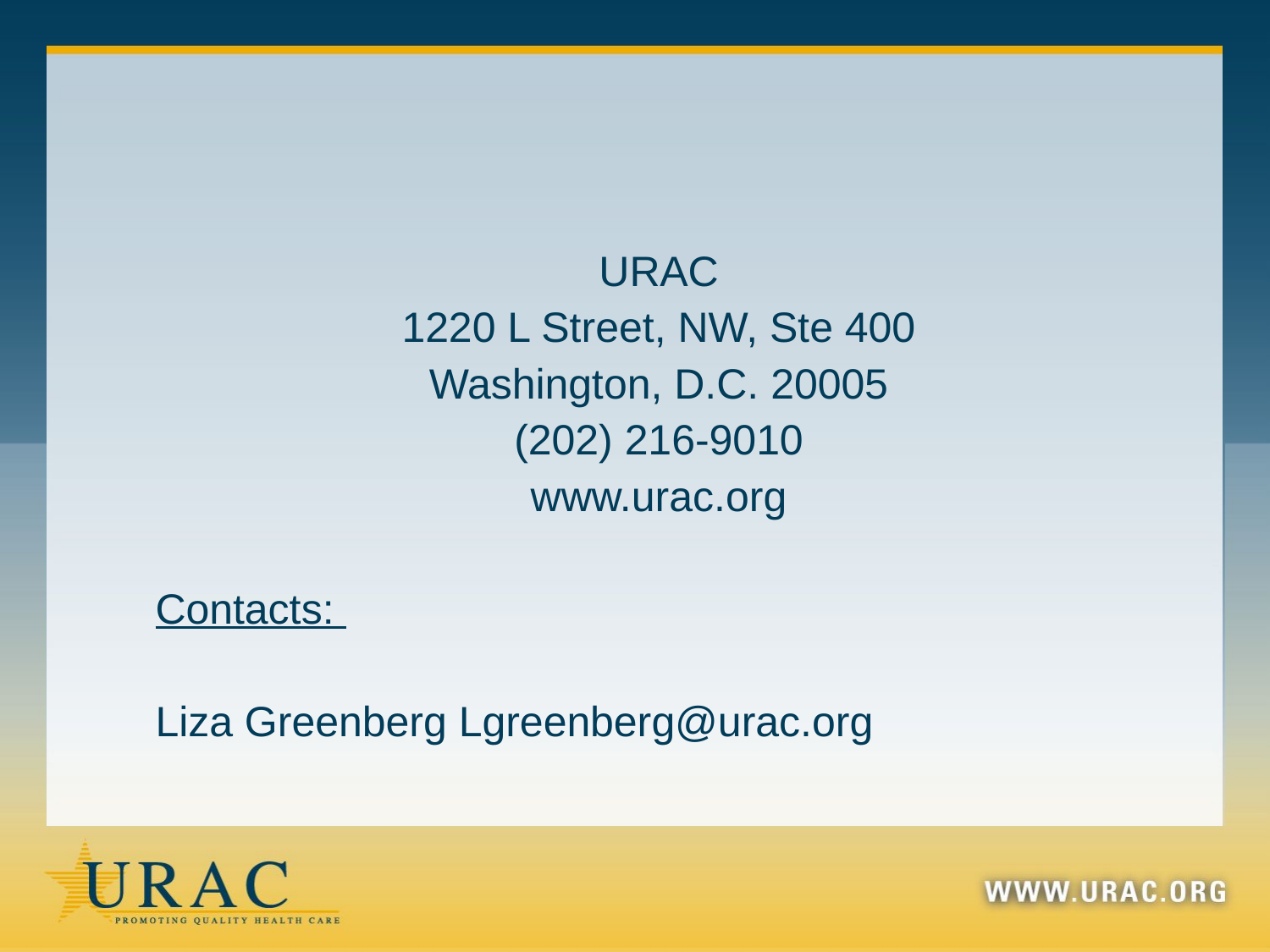

#
URAC
1220 L Street, NW, Ste 400
Washington, D.C. 20005
(202) 216-9010
www.urac.org
Contacts:
Liza Greenberg Lgreenberg@urac.org
